# Cutaneous Disorders Masking Celiac Disease: Case Report and Mini Review with Proposal for a Practical Clinical Approach

**DOI:** 10.3390/nu16010083

**Published:** 2023-12-26

**Authors:** Silvana Ancona, Silvia Bianchin, Noemi Zampatti, Valentina Nosratian, Carolina Bigatti, Jacopo Ferro, Chiara Trambaiolo Antonelli, Gianmaria Viglizzo, Paolo Gandullia, Federica Malerba, Marco Crocco

**Affiliations:** 1 Pediatric Gastroenterology and Endoscopy Unit, IRCCS Istituto Giannina Gaslini, 16147 Genoa, Italypaologandullia@gaslini.org (P.G.); federicamalerba@gaslini.org (F.M.); 2 Department of Neuroscience, Rehabilitation, Ophthalmology, Genetics, Maternal and Child Health (DINOGMI), University of Genova, 16132 Genoa, Italy; 3 Allergy Center, IRCCS Istituto Giannina Gaslini, 16147 Genoa, Italy; 4 UO Nephrology Dialysis and Transplant, IRCCS Istituto Giannina Gaslini, 16147 Genoa, Italy; 5 Pathology Unit, U.O.C. Anatomia Patologica, IRCCS Istituto Giannina Gaslini, 16147 Genova, Italy; jacopoferro@gaslini.org (J.F.); chiaratrambaioloantonelli@gaslini.org (C.T.A.); 6 Dermatology Unit, IRCCS Istituto Giannina Gaslini, 16147 Genoa, Italy

**Keywords:** celiac disease, dermatitis herpetiformis, skin, gluten-free diet, connective tissue disease, vasculitis, acrodermatitis entheropatica

## Abstract

Celiac disease (CD) is an immune-mediated systemic gluten-related disorder characterized by a wide spectrum of intestinal and extra-intestinal manifestations, including damage to cutaneous and connective tissue. We report a rare case of chronic severe dermatitis involving connective tissue and cutaneous vascular vessels as the main clinical presentation of undiagnosed seronegative gluten disorder. A gluten-free diet dramatically improved the intestinal and cutaneous clinical damage in the patient. Pitfalls and the steps of differential diagnosis are described. We also review the literature regarding studies of CD and connective tissue diseases to extend the knowledge of these rare associations. We propose a practical diagnostic approach in suspected CD in autoimmune cutaneous disorders.

## 1. Introduction

The term gluten-related disorders (GRD) refers to a spectrum of chronic disorders triggered by the ingestion of gluten, including celiac disease (CD), wheat allergy, and non-celiac gluten sensitivity (NCGS). 

Celiac disease is a chronic inflammatory disorder of the small bowel that occurs in genetically susceptible individuals [1]. The diagnosis of CD is based on clinical and serological data; however, in adults, histological diagnosis is mandatory according to current guidelines [2,3]. Multiple biopsies of distal duodenum (at least four) and biopsies of the duodenal bulb (at least one, more in case of endoscopic evidence of CD) should be performed due to the possibility of patchy lesions [4] and ultra-short disease [5,6]. The HLA determination is not mandatory but might help doctors exclude CD in atypical cases due to its high negative prognostic value [2]. Less than 5% of CD patients are seronegative, i.e., present the impossibility of identifying classic serological biomarkers of CD [7]. Celiac disease has a wide spectrum of clinical manifestations: in addition to the classic gastrointestinal symptoms, it can affect a wide range of extraintestinal organs, including the skin [8]. Dermatitis herpetiformis (DH) is a gluten disorder characterized by a pruritic vesicular rash triggered by dietary gluten and characterized by deposits of immunoglobulin A (IgA) at the tips of the dermal papilla, which affects the extensor surfaces of elbows, knees, buttocks, and scalp [8]. DH is the dermatological disease most frequently associated with CD, and its treatment is a strict gluten-free diet (GFD). However, in the literature, an increased risk of other skin disorders is reported in CD, including psoriasis [9,10,11,12,13,14], alopecia areata (AA), urticaria [15,16], and vitiligo [17,18,19]. CD can also present with cutaneous signs overlapping with connective tissue disease (CTD), cutaneous vasculitis (CV), or other rare dermatological signs of malabsorption, making it more difficult to diagnose. 

Herein, we present a challenging case of chronic acrodermatitis in a 53-year-old woman as the main presenting symptom of seronegative celiac disease (SNCD). We review the literature regarding previously reported cases of CD and CV or CTD to extend the knowledge of these rare associations. We propose a practical diagnostic approach including screening strategies of CD in cutaneous autoimmune disorders.

## 2. Case Report

A 53-year-old Italian woman with a long history of chronic eczema that presented with worsening acrodermatitis and intermittent diarrhea was referred by her new general practitioner (GP) to our CD center for a second medical opinion regarding the SNCD diagnosis for which a GFD had been prescribed by another center. 

Since her childhood, she had suffered from a severe form of dermatitis classified as atopic. Recurrent abdominal pain, heartburn, and reflux-like symptoms began to occur during adolescence. At 20 years old, she began to report occasional itchy cutaneous eruptions. After her first pregnancy, at 33 years of age, a worsening of dermatitis occurred, with severe desquamation and episodes of Raynaud’s phenomenon involving both hands and feet. Autoimmune thyroiditis was also diagnosed, and treatment was started. Five years later, she developed occasional episodes of urticaria, brown or livid nodules, and edema in the lower limbs. At intervals, over almost 8 years, her GP prescribed treatment with oral steroid without any significant improvement. In subsequent years, colchicine and antihistamines were prescribed by her dermatologist; however, they were completely unsuccessful, and the skin disease continued to worsen.

At 51 years old, she experienced a worsening of acrodermatitis, especially on her feet, and she began to report fatigue and intermittent diarrhea. 

A new dermatologic examination found erythematous desquamative patches, erosions, and crusted lesions involving feet and distal legs. At that time, the patient was not on any medication. The rest of the patient’s physical examination was unremarkable. In her family history, she reported psoriatic arthritis in her mother and undifferentiated connective tissue disease (UCTD) in her sister. 

Routine blood tests were performed: a mild iron deficiency anemia (Hb 10.9 g/dL, normal range (n.r.) 11.5–16 g/dL; MCV 78.6 fL, n.r. 80–100 fL; ferritin 6.9 ng/mL, n.r. 8–252 ng/mL; iron 23 µg/dL, n.r. 50–170 µg/dL) and hypovitaminosis D (25-hydroxy vitamin D 9.6 ng/mL, n.r. 30–100 ng/mL; PTH 116 ng/mL, n.r. 11–67 ng/mL) were found. The patient’s liver and kidney function, albumin, vitamin B12, folate, and coagulation were normal, and her blood inflammatory markers were negative. Elevated fecal calprotectin was found (152 µg/gr), while the testing for Giardia and Helicobacter pylori antigen in the stool resulted negative. Viral infections were also excluded (HIV, Parvovirus B19, and Herpes Simplex Virus 1 and 2). Further rheumatologic investigations were performed based on the hypothesis of vasculitis or autoimmune diseases involving the connective tissue. The rheumatoid factor (RF) and complement factors (C3 and C4) resulted normal, while cryoglobulins were negative. An extended autoantibody panel was performed: antinuclear (ANA), extractable nuclear antigen (ENA), anti-smooth muscle (ASMA), anti-double stranded DNA (anti-dsDNA), anti-mitochondrial (AMA), anti-cardiolipin (aCL), β_2_ glycoprotein 1 (β_2_GPI), and anti-neutrophil cytoplasmic antibodies (ANCA) all resulted negative. The human leukocyte antigen (HLA) B27 was negative. 

Because of the recurrent episodes of diarrhea and the presence of mild anemia, she underwent CD screening: both IgA anti-tissue transglutaminase antibodies (tTG-IgA, ELISA kit) and anti-endomysial antibodies (EMA) resulted negative, with normal total IgA levels, while the antibodies against deamidated gliadin peptide IgG (anti-DGP) were slightly positive (12 U/mL, nr. <7–10). The patient resulted positive to HLA DQ2 (DQB1*0202, DQA1*0201, DRB1*07). Due to clinical persistence of the gastrointestinal symptoms, an endoscopic evaluation was undertaken, showing macroscopic normal duodenal mucosa; unfortunately, no biopsies were performed. 

A punch biopsy of the left foot showed hyperparacheratotic, acanthotic, and spongiotic epidermis and moderate lymphohistocytic and granulocytic perivascular infiltrate in the dermis: these findings were consistent with a diagnosis of dermatitis with non-specific histological features of cutaneous inflammatory disease. Unfortunately, direct immunofluorescence was not performed. 

One year later, due to the persistence of the cutaneous signs and abdominal symptoms (abdominal pain and diarrhea), a serologic screening for CD was repeated, again resulting negative. In agreement with the patient, a second endoscopic evaluation was performed. Due to a clinical suspect of SNCD, although the macroscopic appearance of the duodenal mucosa was apparently normal, five duodenal biopsies were performed and orientated. The histological evaluation revealed focal (patchy) areas of crypt hyperplasia, increased number of intraepithelial T-lymphocytes CD3+ (approximately 30/100 enterocytes), and short villi, consistent with grade II of the Marsh–Oberhuber classification. Based on clinical and histological data, a gluten disorder (such as SNCD) with a significant skin involvement was suspected and a GFD challenge was prescribed. 

**Figure 1:**
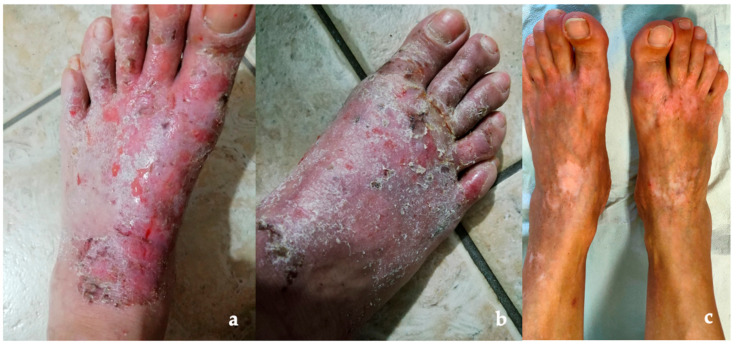
Chronic dermatitis of the patient’s feet with dermographism and urticaria factitia, on the left (**a**) and on the right (**b**). Visible improvement of the patient’s lesions on her feet after GFD, with residual dyschromia and scarring (**c**).

Within three months of the GFD, complete resolution of her gastrointestinal symptoms and cutaneous signs were reported (Figure 1), and her blood tests, including hemoglobin, iron balance, and vitamin D, normalized. The clinical remission was very rapid and impressive, with a significant improvement of quality of life and fatigue; this recovery induced the patient to refuse a second endoscopy during the GFD (to document mucosal healing) to confirm the diagnosis of SNCD, as well as a new skin biopsy after the gluten challenge to exclude an overlapping DH. 

This case report is a rare example of chronic severe dermatitis involving connective tissue and cutaneous vascular vessels as the main clinical presentation of undiagnosed gluten disorder. After many years of investigation and various unsuccessful immunosuppressive treatments, a diagnosis of SNCD was hypothesized and a challenge with a GFD was initiated. A subsequent rapid and dramatic improvement of both cutaneous and gastrointestinal symptoms occurred and has been maintained. Currently, the patient has a good quality of life without any skin or gastrointestinal disorders. The patient refused a gluten challenge as proposed after our case evaluation. Therefore, after discussion with the pathologist about the correct orientation and re-evaluation of duodenal biopsies (skin biopsy was not available) we confirmed potential “patchy” SNCD with severe clinical expression and a good response to the GFD. 

## 3. Review: CD Association with Connective Tissue Diseases and Cutaneous Vasculitis

**Figure 2:**
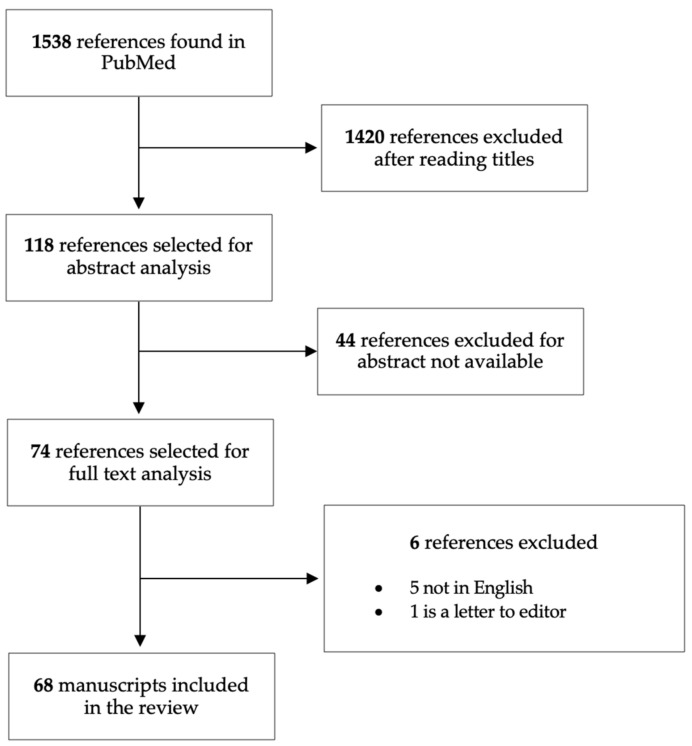
Flow chart of the identified and selected studies.

**Figure 3:**
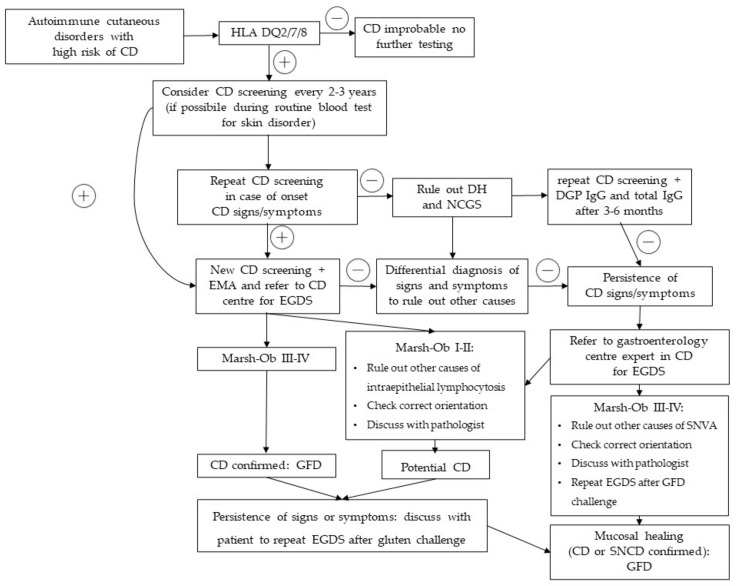
CD Screening strategy and practical diagnostic approach in suspected CD in cutaneous autoimmune disorders. + = positive or present − = negative or not present.

In order to extend the knowledge about the occurrence of CD in autoimmune cutaneous disorders involving connective tissue and/or vasculitis, authors S.A. and S.B. reviewed the literature from the last five decades. Searches were undertaken in the PubMed database in February 2023 using a combined search of the following terms: “celiac disease” AND “skin”, “celiac disease” AND “systemic lupus erythematosus”, “celiac disease” AND “systemic sclerosis”, “celiac disease” AND “Sjogren’s syndrome”, “celiac disease” AND “undifferentiated connective tissue disease”, “celiac disease” AND “dermatomyositis”, “celiac disease” AND “cutaneous vasculitis”. We also reviewed the references within the articles to identify additional sources. Only articles in English were considered. A total of 1538 articles were found this way, 69 of which were selected, as reported in Figure 2. Finally, we propose a practical diagnostic approach including screening strategies of CD in cutaneous autoimmune disorders (Figure 3). 

### 3.1. CD Skin Manifestations Overlapping with CTD

The association of CTD and CD has been reported in the literature [20,21,22], and genetic predisposition seems to play an important role. Among CTD, dermatomyositis, a rare autoimmune disease which typically affects the skin, the muscles, and the blood vessels, is the most common CTD described in association with CD [23,24,25,26]. In patients with coexisting CD and DM, a GFD may improve the cutaneous signs of DM [27].

As for systemic lupus erythematosus (SLE), Ludvigsson et al. suggested that individuals with CD have a 3-fold risk of SLE compared to the general population [28]. A recent study by Soltani et al. reported a prevalence of 3% for biopsy-proven CD in patients with SLE [29], while Shamseya et al. found biopsy-confirmed CD in 6% of juvenile SLE population [30]. Another cutaneous disorder in SLE is chilblain, also known as lupus pernio. It is usually characterized by painful papuloerythematosus plaques on the fingers due to superficial and localized inflammation resulting from a maladaptive vascular response to non-freezing cold. To the best of our knowledge, there are only three reported cases in children, all characterized by a significant improvement after adopting a GFD [31,32,33].

The association between Sjogren’s syndrome (SS) and CD has been reported in several case reports [34,35,36]. However, the prevalence of CD in patients with SS is not clear, ranging from 1% to 15% [37,38,39,40], and, in recent research, SS occurrence in CD patients varies from 1.2% to 6.5% [41,42,43,44]; this discrepancy may be due to a gap in the diagnosis of CD in SS patients. The link between CD and SS is supported by research studies that demonstrate GFD effectiveness in the control of SS symptoms in patients affected by both diseases [45]. 

A few publications have reported the coexistence of CD and systemic sclerosis (SSc) [46,47] with a prevalence from 4% to 8% [48,49,50], though the association between these two conditions remains controversial [51]. There is also a study that suggests a higher prevalence of CD in UCTD compared to the general population [52].

Among the overlapping symptoms, AA is an autoimmune disease common in both CD and CTD, especially SLE. The risk of alopecia is three times greater in patients with CD than in the general population [53]. Although its etiopathogenesis is still unclear, a T-cell-mediated reaction has been recognized [54], and AA may improve after starting a GFD in CD patients [55,56]. Moreover, both chronic urticaria (CU) and sclerodactily have been identified as dermatological manifestations of CD [15,16,57,58]. In a study, the odds ratio of having CD was 26.9 in patients with CU (95% CI, 6.6–110.17; *p* < 0.0005), compared to the control subjects [59]. Therefore, in cases of CU, CD screening should be suggested [60,61].

### 3.2. CD Skin Manifestation Overlapping with Cutaneous Vasculitis

Cutaneous vasculitis is an inflammatory process affecting the dermal blood vessel wall and leading to its destruction with subsequent ischemic and hemorrhagic events. CV is generally characterized by petechiae, palpable purpura, and infiltrated erythema [62]. The association between CD and CV has been reported in several studies [63], and the literature suggests that CV is more likely to occur in patients with poorly controlled CD and that a GFD may improve CV lesions in such cases [64,65]. 

### 3.3. Acrodermatitis Entheropatica Secondary to CD

A typical feature of CD is the malabsorption and subsequent deficiency of micronutrients. Among these, zinc deficiency is the most common, and it causes alopecia as well as erythematous-squamous dermatitis in the periorificial regions, genitals, and arm flexures. However, a few cases of acrodermatitis entheropatica (AE) secondary to CD have been described in the literature [66]. In these patients, cutaneous manifestations improve with a GFD and oral zinc supplement. 

## 4. Discussion

Celiac disease is a chameleonic disease [67]. It may be associated with various cutaneous disorders, ranging from the most common DH to rare cases of severe chronic dermatitis which may hide CD. 

In our case report, the dramatic clinical improvement of the patient’s cutaneous lesions after the initiation of a GFD allowed us to hypothesize that the connective tissue and cutaneous vascular vessels damage constituted the main clinical presentation of undiagnosed SNCD. Although Marsh–Oberhuber II lesions cannot be classified as CD lesions [68], the patient’ symptoms and their extraordinary clinical response to the GFD support the assumption that this patient is affected by a gluten disorder and that the treatment with a GFD seems to be justified. Therefore, we offered the patient a follow up for the surveillance of high risk of complications in the SNCD [7,69,70].

In the case reported, the investigations to exclude an autoimmune gluten disorder started after several years of diagnostic delay. This is not surprising as CD remains largely underdiagnosed and is still burdened by a severe diagnostic delay, which may have been worsened by the SARS-CoV-2 pandemic [71,72]. Due to the complexity and heterogeneity of CD, it is difficult to include it in a “rigid” diagnostic algorithm covering all clinical aspects. For this reason, a quantitative approach was proposed by Catassi and Fasano to help clinicians avoid misdiagnosis [73]. Using this method, the diagnosis of CD is confirmed if at least four of the following five criteria are fulfilled: typical symptoms of CD; positivity of serum CD IgA class autoantibodies; HLA-DQ2 and/or HLA-DQ8 genotypes; celiac enteropathy found on small bowel biopsy; and response to a GFD. In our case, the “4 out of 5” rule was satisfied; therefore, a SNCD diagnosis could be confirmed. 

Patients with lesser degrees of villous atrophy are less likely to have positive CD serologies [74,75,76]. In Aziz et al.’s work on a prospective UK center experience evaluating 200 adults with seronegative villous atrophy, only 62 cases were identified as SNCD. Of them, 48 patients were diagnosed on the basis of having positive HLA-DQ2 and/or DQ8 status, no alternative causes found, and villous atrophy following a gluten challenge, with a subsequent clinical and/or histological response to a GFD; a total of 14 patients were diagnosed based on selective IgA deficiency with raised IgG celiac serology (*n* = 9) or first-degree family history of CD alone, with subsequent response to a GFD (*n* = 4); one patient had dermatitis herpetiformis. The histological grading of duodenal biopsies of SNCD showed intraepithelial lymphocytosis in 98.4% of cases, with the majority of patients found to have partial villous atrophy (62.9%). In contrast, subtotal villous atrophy was seen in 24.2% of the patients and total villous atrophy in only 12.9% of cases [69]. The anti-DGP was not evaluated in this study. 

In our case, a slight positivity of anti-DGP was found with a low title as the only autoantibody of CD. Anti-DGP positivity is recognized in some CD cases with a negative TTG IgA testing and normal IgA [77] and can represent an early preclinical biomarker predicting CD onset [78]. Moreover, anti-DGP have potential advantages over other conventionally used assays in milder forms of enteropathy associated with gluten-sensitive skin disorder and have been detected more frequently and at higher serum concentrations in patients with autoimmune skin disorders compared to healthy controls [79]. However, an isolated increase in anti-DGP in patients with negative conventional CD serological testing has a very low predictive value [77]. 

Considering the TTG IgA and EMA seronegativity in the patient, the impossibility of evaluating the histological response to the GFD and/or gluten challenge represents an important limitation to definitive conclusion in the reported case. Unfortunately, in this case, the patient refused to undergo either a second endoscopic evaluation while following a GFD to confirm SNCD or a rechallenge with gluten reintroduction to perform skin biopsy to rule out other gluten disorders such as DH. Furthermore, other limits of our case are the fact that the cutaneous biopsy was carried out without immunofluorescence and that the zinc levels were unknown before the initiation of the GFD, so DH and acrodermatitis entheropatica cannot be definitively excluded. 

Villous atrophy is present in several conditions [80,81,82], so it is mandatory that every histological finding of seronegative villous atrophy is included in a differential diagnosis with other non-celiac enteropathies to avoid misdiagnosis. Classic serological CD screening may not be sufficient to exclude SNCD, and a gluten challenge is still necessary to confirm the clinical suspicion of SNCD [83,84]. In these rare cases, intestinal deposits of EMA or TTG can be identified using immunofluorescence (also ELISA for TTG) on frozen sections [85,86]. The new method based on a double immunohistochemistry for the determination of intestinal TTG IgA deposits on formalin-fixed paraffin-embedded biopsies [87] could help the diagnosis in case of the unavailability of frozen tissue. Anti-TTG are produced and can be detected in the small bowel before they appear in circulation [88]. 

SNCD not only involves the gut, but it is a systemic disease, often involving the skin, and it should be taken into account in the workup of cases of gluten-related disorders [89]. On the other hand, cutaneous manifestations may be the only sign of an autoimmune disease including CD. To date, various and polymorphous cutaneous manifestations have been identified as being associated with CD. The IgA autoantibodies against TTG type 3 have been reported to be more predictive than TTG type 2 in patients with DH [90]. EMA antibodies were first discovered by Chorzelski et al. in patients with DH [91]. The EMA test is a subjective semiquantitative method of immunofluorescence, currently used as a confirmatory test in CD, although burdened by possible false-negative results and a risk of not detecting villous atrophy in CD [92,93]. Patients with DH and other cutaneous gluten-dependent disorders may be EMA- and/or TTG-seronegative [94,95]. The presence of skin manifestations should alert the clinicians to the possibility of CD, which should be included in a differential diagnosis workup, even in the absence of digestive or malabsorptive symptoms. Indeed, beyond the best-known and frequently described DH, also known as the “CD of the skin”, CD may appear with a wide range of other autoimmune skin manifestations, including stigmas of CTD or vasculitis. Finally, CD can be hidden by skin disease associated with malabsorption and deficiency of different micronutrients and oligoelements, in particular zinc [96].

In our review of the literature, CD prevalence in some autoimmune diseases (e.g., CTD as SS) is higher (up to 15%) than in the general population [37,38]. Nevertheless, few data are available, in particular regarding the prevalence, prognosis, and management of these diseases. For some dermatological pathologies, data are still limited to small case series demonstrating poor understanding of the problem. The patients with CD are at an increased risk of multiple common skin disorders compared to the general population, as evaluated over the long term by Lebwohl et al. [97] in 43,000 CD patients and 198,000 controls. 

Various hypotheses have been proposed to explain the link between CD and cutaneous manifestations. A leakage of intestinal permeability is reported in several dermatological diseases. The loss of the intestinal barrier may allow the exposition of exogenous antigens triggering the immunological system, which would lead to vascular alterations which would perpetuate skin and intestinal damage. However, only a few associations between CD and skin diseases (e.g., DH and psoriasis) are based on strong levels of evidence. The other associations are based, often, on small case series and a few controlled studies (Table 1) [19].

The CD screening strategy in autoimmune skin disorders is not yet applied systematically in clinical practice. Presently, the recommendations on “who should be tested for CD” guidelines exclude cutaneous manifestations from the list of indicated “warning signs” [109,110]. However, considering the high prevalence of CD in autoimmune diseases involving the skin (which emerges from the limited available literature reviewed), a screening test should be included in the workup of refractory cutaneous manifestations, as recent guidelines have suggested for CU in children [61]. To reduce the “ice-berg” of unidentified CD, we propose a practical diagnostic approach (Figure 3) including screening strategies of CD in cutaneous autoimmune disorders, similar to the one recommended in other autoimmune diseases [68,111].

Evaluating the other pitfalls of the reported case, the patchy distribution of histological damage increased the complexity of diagnosis. There are anecdotal cases of patchy distribution of villous atrophy in adults with dermatitis herpetiformis in which areas of mucosal atrophy may be adjacent to normal duodenal mucosa [112]. Patchy villous atrophy has been well characterized in children and adults with CD, in whom atrophy may be also limited to the duodenal bulb with normal histology in the distal duodenum [4,113,114]. The patchy distribution of gluten-sensitive enteropathy, particularly when limited to the bulb, is linked to a low title of serological markers of CD and underdiagnosis if few specimens of normal mucosa are taken during endoscopy [115]. Moreover, due to a self-fulfilling mechanism, SNCD is likely to be underestimated due to the tendency to perform small intestinal biopsy only in patients with positive CD serum markers [74]. Physicians need to maintain a high degree of clinical suspicion in these rare cases that should be referred to specialized CD centers. However, the diagnosis and management of SNCD is still a clinical challenge and requires a close collaboration between clinician, pathologist, and patient. There is a need for additional studies to increase the knowledge of the potential impact of gluten disorders on different cutaneous conditions and the potential therapeutic effect of a GFD.

## 5. Conclusions

Skin diseases represent a common extra-intestinal manifestation of CD. Gluten disorders are still a diagnostic and therapeutic dilemma. Individuals with a suspected skin manifestation of SNCD should not be prescribed a GFD before concluding the mandatory investigation to rule out seronegative non-celiac diseases. These challenging cases should be managed from the beginning by tertiary referral centers to reduce diagnostic pitfalls and avoid unnecessary treatment. In cases of skin lesions resistant to standard therapies, a correct diagnostic approach may help doctors identify these conditions earlier. The practical diagnostic approach proposed in this paper delves deeper into the submerged “celiac iceberg” in an attempt to identify patients with autoimmune skin disorders who may benefit from a gluten-free diet, even in the absence of gastrointestinal symptoms and abnormal celiac serology.

## Figures and Tables

**Table 1:** Overview of the reviewed sources. JIA—juvenile idiopathic arthritis; JSLE—juvenile systemic lupus erythematosus; SLE—systemic lupus erythematosus; SS—Sjogren’s syndrome; pSS—pediatric Sjogren’s syndrome; SSc—systemic sclerosis; UCTD—undifferentiated connective tissue disease; DM—dermatomyositis; CV—cutaneous vasculitis; CU—chronic urticaria; DM1—type 1 diabetes mellitus; DH—dermatitis herpetiformis; GFD—gluten-free diet; RA—rheumatoid arthritis; y—years; and pt—patient.

Authors	Year	Population	Primary Objective	Results
Doe WF et al. [98]	1972	*n* = 4 adult patients (M 42 y, F 31 y; F 67 y; and M 50 y)	To evaluate four cases of CD, vasculitis, and mixed cryoglobulinemia	
Marguerie C et al. [47]	1995	*n* = 2 (a 35-year-old Caucasian woman and a 40-year-old man)	To describe two cases of concomitant scleroderma and CD	
Iltanen S et al. [37]	1999	*n* = 34 (pSS): median age 55 y, range 26–70; F 82% *n*= 28 (healthy controls): median age 53 years, range 25–81; F 43%	To evaluate the prevalence of CD in patients with pSS	Prevalence of CD was 14,7% in the pSS patients
Rensch MJ et al. [99]	2001	*n* = 103 (SLE)	To evaluate the prevalence of CD autoantibodies in SLE patients	23.3% of the SLE patients tested positive for AGA, whereas none tested positive for EMA nor had histological evidence of CD
Luft L et al. [49]	2003	*n* = 50 (SS) *n* = 50 (SLE) *n* = 30 (RA) *n* = 30 (SSc) *n* = 50 (healthy controls)	To evaluate the prevalence of anti-tTG in a cohort of SS patients and other systemic rheumatic diseases	Prevalence of anti-tTG: SS 12% vs. heathy controls 4% vs. SLE 6% vs. SSc 7% vs. RA 2%. A total of 5/6 SS patients with anti-tTG had symptoms, signs, or small bowel biopsy findings consistent with a diagnosis of CD
Szodoray P et al. [100]	2004	*n* = 111 (SS)	To evaluate the prevalence of CD in patients with SS	Prevalence of CD: SS patients 4.5% vs. 0.45–0.55% in non-SS population
Patinen P et al. [45]	2004	*n* = 20 (CD and SS): age mean 61 years, range 48–78; F 95%	To evaluate the prevalence of oral mucosal and dental abnormalities in patients with SS and CD	Prevalence of oral mucosal abnormalities: SS 80% vs. CD + SS 65% vs. CD 40%. The CD + SS patients had higher salivary flow rates and lower inflammatory focus scores in the salivary glands than the SS patients
Gabrielli M et al. [101]	2005	*n* = 80 (CU): age mean 48 ± 18 y; F 72.5% *n* = 264 (healthy controls): age mean 45 ± 16 y; F 72.7%	To evaluate the prevalence of CD in a population of adult patients with CU and healthy controls	No differences in CD prevalence in the two groups (1.25% in the CU group vs. 0.38% in the healthy controls)
Caminiti L et al. [15]	2005	*n* = 79 (CU): median age 7 y, range 2–18; F 51.9%	To evaluate the prevalence of CD in children with urticaria	CD prevalence: CU 5.0% vs. healthy controls 0.67%; (*p* = 0.0003)
Seyhan M et al. [102]	2007	*n* = 55 children and adolescents (mean age 10 ± 4.6 years; F 58.2%)	To evaluate the prevalence of mucocutaneous manifestations of CD in childhood and adolescence	The most prevalent dermatologic diagnosis was xerosis (69.1%)
Freeman HJ et al. [103]	2008	*n* = 246 (biopsy-defined CD)	To evaluate the prevalence of SLE in CD patients	SLE occurrence in the CD patients was higher than in the general population (2.4% biopsy-proven CD)
Rosato E et al. [50]	2009	*n* = 50 (SSc): mean age 51 ± 14.5 y; F 86%	To evaluate the incidence of CD in patients with SSc	The incidence of CD in the patients with SSc was 8%
Nisihara R et al. [51]	2011	*n* = 105 SSc Brazilian patients (mean age 43.2 years; F 92.3%)	To evaluate the prevalence between CD and SSc	No association between CD and EMA positivity
Ludvigsson JF et al. [28]	2012	*n* = 29,048 (biopsy-verified CD): median age 30 y, range 0–95; F 61.9% *n* = 144,352 (healthy controls)	To evaluate the prevalence of SLE in patients with CD	The CD patients were at a 3-fold increased risk of SLE compared to the general population (HR 3.49%; 2.48–4.90)
Forbess LJ et al. [48]	2013	*n* = 72 (SSc): mean age 51 ± 13 y; F 88%	To evaluate the prevalence of CD in SSc patients	The prevalence of CD in the SSc patients was 4%
Ludvigsson JF et al. [16]	2013	*n* = 28,900 (biopsy-verified CD): median age 30 years, range 0–95; F (61.8%) *n* = 143,397 (healthy controls): median age 30 years, range 0–95; F 61.9%	To evaluate the risk of urticaria in CD patients	The patients with CD were at an increased risk of later urticaria (HR = 1.51; 95% CI = 1.36–1.68 based on 453 observed events vs. 300 expected in CD patients without a diagnosis of earlier urticaria) The patients with CD were at an almost two-fold increased risk of chronic urticaria (HR = 1.92; 95% CI = 1.48–2.48, based on 79 observed events vs. 41 expected)
Conti V et al. [52]	2015	*n* = 52 (UCT): median age 44 years, range 21–69; F 98%	To evaluate the prevalence of CD in UCTD	Prevalence of CD in UCTD was 11.5%
Ludvigsson JF et al. [104]	2018	*n* = 29,096 (CD) *n* = 144,522 (healthy controls) (F 62% in both groups)	To evaluate the prevalence of HSP in CD patients	No association between CD and HSP (0.06% in the CD patients vs. 0.07% in the controls; HR 0.96)
Soylu A et al. [105]	2016	*n* = 42 (children with HSP): mean age 126 ± 49 months; F 40%	To evaluate CD prevalence in children with HSP	CD seropositivity prevalence: HSP 12% vs. Turkish school children 2.5% (*p* < 0.001)
Erbasan F et al. [43]	2017	*n* = 82 (CD): age mean 40.5 y, range 20–62; F 73%	To evaluate the prevalence of SS in CD patients	The prevalence of dry-eye symptoms was 29.3%, but nine patients (11%) were using systemic medications that could contribute to their dry-eye symptoms
Caio G et al. [40]	2018	*n* = 230 (67 RA, 52 SS, 42 SSc, 35 SLE, 15 MCTD, 11 PM, and 10 DM): age range 18–84 y; F (81.7%)	To evaluate the prevalence of CD seropositivity in a cohort of patients referred to an Italian rheumatological clinic	CD (antibodies) prevalence: rheumatological patients: 3% (in SS 5.8%; in SSc 8%; in RA 1.5%; and in SLE 2.8%)
Bartoloni E et al. [39]	2019	*n* = 580 (SLE): age mean 46 ± 13 y; age range 19–83; F 89% *n* = 354 (pSS): age mean 55 ± 12 y; age range 21–90; F 97% *n* = 524 (SSc): age mean 61 ± 14 y; age range 15–87; F 90% *n* = 14,298 (healthy controls): age mean 53 ± 22 y; age range 15–90; F 91%	To evaluate CD prevalence in SLE, pSS, and SSc	CD prevalence: pSS 6.8% vs. controls 0.6%, *p* < 0.0001); SLE (1.4%, *p =* 0.058) and SSc (1.3%, *p =* 0.096)
Sahin Y et al. [106]	2019	*n* = 50 (JSLE) (age mean 15.5 ± 3.4 y; F 88%)	To evaluate the prevalence of CD in children with SLE	No CD in children with SLE
Kosmeri C et al. [107]	2019	*n* = 49 (children with CU): median age 8.8 years, range 1–15; F 46.9%	To evaluate the association between autoimmune diseases and CU in children	The prevalence of CD biopsy-confirmed in CU was 2%. The prevalence of high serum levels of anti-thyroid antibodies but normal thyroid function was 8.1%. No other specific autoantibodies were detected
Shamseya AM et al. [30]	2020	*n* = 100 (JSLE): age mean 34.6 ± 9.6 y; age at diagnosis 11.9 ± 3.4 y; F 90% *n* = 40 (healthy controls): age mean 35.5 ± 9.3 y; F 87.5%	To evaluate the prevalence of CD in patients with JSLE	Positive serology in 10% of the SLE patients (vs. 0% in the controls), biopsy-confirmed CD in 6%
AlEnzi F et al. [108]	2020	*n* = 81 (SL): age mean 34.1 ± 11.5 y; F 94% *n* = 34 (JSLE): age mean 10.3 ± 2.7 y; F 82% *n* = 62 (RA): age mean 48.8 ± 10 y; F 94% *n* = 73 (JIA): age mean 10.0 ± 2.6 y; F 62%	To evaluate the prevalence of CD in adults and children with SLE and compare them with RA and JIA	The prevalence of serologic CD positivity was higher (but not statistically significant, *p =* 0.27) among the SLE patients (13.1% vs. 10.4% among the RA and JIA patients), but nobody had biopsy-proven CD
Ayar K et al. [42]	2020	*n* = 80 (CD): age mean 40.5 ± 13.5 y *n* = 100 (healthy controls): age mean 39.7 ± 12.5 y	To evaluate the prevalence of sicca symptoms and SS in CD patients	Prevalence of ocular symptoms: CD 22% vs. healthy controls 13%, (*p* = 0.113); oral symptoms: CD 26% vs. healthy controls 10%, (*p =* 0.005); SS CD: 2.8% according to ACR criteria and 5.0% according to AECG vs. healthy controls 3.0% and 2.0%
Lebwohl B et al. [97]	2021	*n* = 43,300 (CD): age mean 31.3 ± 25.2 y; F 62.4% *n* = 198532 (healthy controls): mean age 30.6 ± 25 years; F 62.3%	To evaluate the risk of skin disorders in CD patients	The CD patients had an increased risk of multiple common skin disorders (HR 1.55). Higher risk in the men and in the patients aged 18–40 years; strongest association after DH was vitiligo (OR 2.20), followed by alopecia areata (OR 2.07), psoriasis (OR 1.86), eczema (OR 1.59), and urticaria (OR 1.53)
Soltani Z et al. [29]	2021	*n* = 130 (SLE): age mean 31.5 ± 8.3 y; F 81.5%	To evaluate the prevalence of CD in patients with SLE	Prevalence of 3% for biopsy-proven CD in the patients with SLE

## Data Availability

The data presented in this study are available on request from the corresponding author.
